# Men's Sheds: A conceptual exploration of the causal pathways for health and well‐being

**DOI:** 10.1111/hsc.12765

**Published:** 2019-06-17

**Authors:** Danielle Kelly, Artur Steiner, Helen Mason, Simon Teasdale

**Affiliations:** ^1^ Yunus Centre for Social Business and Health Glasgow Caledonian University Glasgow UK

**Keywords:** causal pathways, health, men, Men's Shed, well‐being

## Abstract

Although men have a lower life expectancy than women, and are more susceptible to illness, they have been found to be less likely to engage in health‐seeking behaviour. Men's Sheds, as a gendered intervention, has been identified as an effective way to engage men in meaningful activity and gain social support from others. However, links between sheds and health and well‐being are not well‐documented, and evidence is lacking of the potential causal pathways to health generation. This study aims to develop a plausible empirically based causal theory of how Men's Sheds influence the health and well‐being of their participants and to set out future research directions to test this theory. Drawing on a scoping review of academic, peer‐reviewed journal articles published between 1990 and 2018, potential causal linkages between shed activity and health and well‐being outcomes are synthesised into a logic model framework. Sixteen relevant peer‐reviewed journal were identified from the academic literature. The data from the articles are predominantly self‐reported, and characterised by small sample sizes and/ or low response rates. Further, information is lacking on the demographics of Men's Shed participants and the contexts in which they exist. Most notably, while there is some evidence on the potential mental health and social well‐being impacts of shed activities, physical health is less documented. The study shows that there is a lack of reliable and systematic evidence of the potential causal pathways between Men's Shed activities and health and well‐being outcomes. In order to address research gaps, further research is required to test and develop the proposed theory and logic model.

**What is known about the topic**

- Men have a lower life expectancy than women, and are less likely to engage in health seeking behaviours.
- Men's Sheds have been identified as a possible source of meaningful activity and social support that could contribute to health.
- Evidence is lacking on the potential causal pathways from Men's Shed activity to health generation.

**What this paper adds**

- Identified studies on Men's Sheds and health are based around small sample sizes and self‐reported data.
- Clear research gaps are present, including the need for contextual‐based data and evidence of physical health impacts.
- Further research is required to address research gaps and to test the proposed logic model.

## 1 INTRODUCTION

Men's health is a particularly overlooked public health issue that is under‐represented in health improvement literature (Baker, 2016; Milligan, Dowrick, & Payne, 2013). While studies have provided evidence of gender‐based preventative measures for the health and well‐being of women, only a few studies describe male‐specific interventions (Baker, 2016; McGeechan, Richardson, Wilson, O'Neill, & Newbury‐Birch, 2016). Globally, men have been found to have poorer health and a lower life expectancy than women, with a higher susceptibility to illness and mortality (Baker, 2016; Office for National Statistics, 2015; WHO, 2016). Illnesses found in men have been related to cardiovascular disease in older age (Jousilahti, Vartiainen, Tuomilehto, & Puska, 1999), alcohol consumption patterns (Gawryszewski & Monteiro, 2014), and poorer mental health and higher suicide rates in unemployed and retired men (Qin, Agerbo, Westergård‐Nielsen, Eriksson, & Mortensen, 2000). Retired and unemployed men are also found to be at a greater risk of social isolation and loneliness than those who are employed (Flood & Blair, 2013; Wilson & Cordier, 2013). With the loss of work can come the loss of colleagues, networks, social support and autonomy (Ormsby, Stanley, & Jaworski, 2010).

Although more predisposed to illness, men are less likely to engage in health‐seeking actions than women, and more likely to participate in risky behaviours, such as excessive alcohol consumption (Baker, 2016; Davies et al., 2000; Mahalik, Burns, & Syzdek, 2007). Men are also more likely than women to delay seeking help for mental health problems, such as therapy or counselling, for fear of scrutiny about their masculinity (Men's Health Forum, 2015). Consequently, men are often classed as a ‘hard to reach’ group for preventative health measures and harm reduction (Kirwan, Lambe, & Carroll, 2013). Research from the United Kingdom (UK) has evidenced the positive health outcomes of ‘gender sensitive’ health interventions, such as male‐targeted sports programmes and mental health promotion (Hunt et al., 2014; Robertson et al., 2014). Nonetheless, the UK policy and practice has not yet engaged with the idea that male biology, attitudes and behaviour may require consideration to create male‐friendly health interventions (Baker, 2016).

One potential solution to men's health provision is the ‘Men's Shed’ model. This community‐based movement emerged in Australia, in the 1990s, as a response to the increasing concerns about men's health (Earle, Earle, & Mering, 1996; Wilson & Cordier, 2013). Sheds are practical communal spaces, typically workshop areas that provide opportunities for men to take part in meaningful social and recreational activities that encourage skill sharing and informal learning (Ballinger, Talbot, & Verrinder, 2009; Crabtree, Tinker, & Glaser, 2017; Golding, Brown, & Foley, 2007; Wilson & Cordier, 2013). Sheds provide access to social support and enable men to gain advice, and share experiences and concerns in an informal and unstructured environment. Sheds are particularly supportive in providing a socially acceptable masculine environment for men to socialise and gain a sense of male identity and belonging from their relations with other men^1^ (Ballinger et al., 2009; Golding, 2015). This alternative health and engagement space is provided for men who might be reluctant to access formal healthcare (Golding, 2015); in particular, those marginalised through mental health issues, unemployment or negative life changes (Wilson & Cordier, 2013; Morgan, 2010). Sheds are found to be predominantly useful for older retired men and those who are not engaged in employment, as a way of maintaining a masculine ‘work‐like’ routine and sense of purpose, and to combat social isolation (Kierans, Robertson, & Mair, 2007; Moylan, Carey, Blackburn, Hayes, & Robinson, 2015; Waling & Fildes, 2017). Furthermore, in Australia, this model has also proved particularly beneficial for war veterans with ongoing physical and psychological health issues (Golding, 2011).

Though the concept of Men's Sheds began in Australia, the shed movement is growing globally, most notably in the UK, Ireland, Canada and New Zealand. Nevertheless, sheds are a relatively unexplored concept in academic literature, and evidence that exists originates from predominantly qualitative studies from Australia, based on small sample sizes and self‐reported data. The links between sheds and health and well‐being outcomes are not well documented; most notably, evidence is lacking in relation to the ‘directions of causality’ between shed activities and the potential resultant health outcomes (Milligan et al., 2013).

The aim of this paper is (a) to *develop a plausible empirically based causal theory of how Men's Sheds influence the health and well‐being of their participants* and (b) to *set out future research directions to test this theory*. Through a review of existing literature on Men's Sheds and health and well‐being, a logic model is used to visualise the plausible pathways from shed activities to health and well‐being. Although systematic reviews of Men's Sheds literature are in existence (Milligan et al., 2013, 2016), in this paper, we update these reviews and through the development of a logic model, provide new insights into the plausible causal pathways which will aid hypotheses development in narrowing down specific gaps in literature that require further exploration, and directions for future research.

## 2 METHODS

A scoping review was conducted between January and May 2018, to identify relevant literature on the health and well‐being impacts of Men's Shed activity.

### 2.1 Data sources

A search strategy was developed using the keywords ‘Men's sheds, sheds, male, health, well‐being’ and all possible combinations of the keywords. Literature was sourced through the electronic databases Emerald Insight, Science Direct, ProQuest, PubMed, Cambridge University Press (online), Oxford University Press (online) and Wiley Online. The inclusion criteria for papers were (a) peer‐reviewed journal articles, (b) papers based on primary data relating to the health and well‐being outcomes of Men's Shed activity, (c) English language papers, and (d) publication date between 1990 and 2018. Exclusion criteria were (a) studies reporting on secondary data and (b) studies reporting on an external intervention outside of the shed (e.g. an external mentoring programme). A formal quality assessment was not undertaken due to the scoping nature of the review (Arksey & O'Malley, 2005).

### 2.2 Analysis and logic model framework nature

A database was used to catalogue the details of the articles identified, including publication information, intervention type, shed inputs, and health and well‐being outcomes described. The next step was to thematically synthesise and interpret this data into a logic model. Findings from the papers were coded and descriptive categories developed based on emerging commonly described themes (Thomas & Harden, 2008). A logic model is defined as ‘a diagram of proposed causal linkages among a set of concepts believed to be related to a particular public health problem’, and is typically used as a framework to organise and integrate information related to a particular research area (Earp & Ennett, 1991:164]. Inputs were defined as activities or processes within a shed that may affect the health and well‐being of individuals involved with the shed (e.g. interaction with others, taking part in woodwork). Outcomes of the activities and processes described were divided into mediating variables, intermediate and long‐term outcomes. *Mediating* variables were used to provide an understanding of the mediating relationship between the input and the health and well‐being outcome (see Figure 2). For example, the provision of woodwork facilities (the input), led to shed members taking part in group woodwork activities alongside others (the mediating variable), which then led to intermediate‐ and long‐term health and well‐being outcomes. *Intermediate* and *long‐term outcomes* were used to describe the short‐ and longer term effects of shed activity and processes on health and well‐being in a visual linear pathway. Key mechanisms that may contribute towards improved health and well‐being in shed members were then identified.

## 3 RESULTS

### 3.1 Paper sample

**Figure 1:**
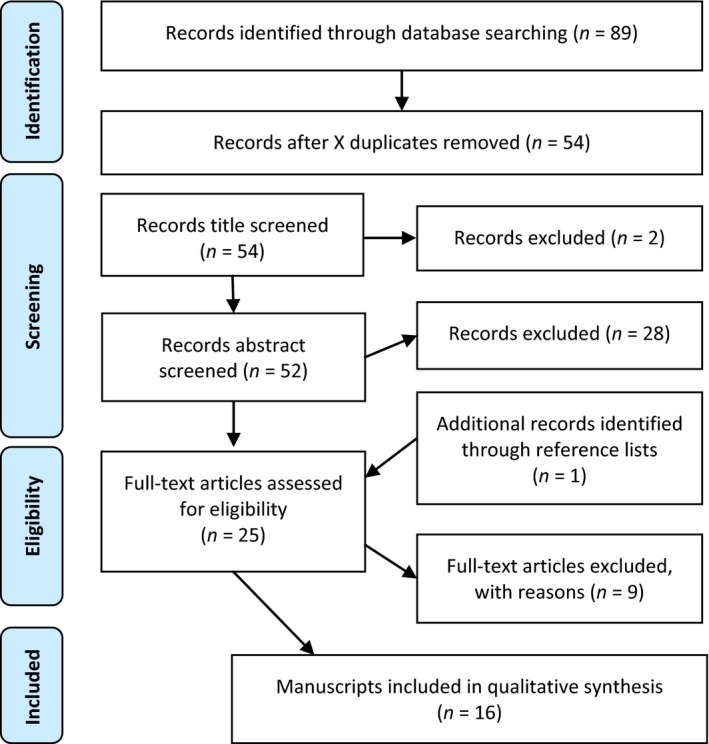
PRISMA flow diagram of article selection process

An electronic search generated 16 papers that were suitable for full review, as shown in Figure 1.

For the results section of this study, each paper will be referred to using an identification reference number, as shown in Table 1. The papers consisted of 11 studies from Australia, 3 from the UK, 1 from Ireland and 1 multicountry study (involving Men's Sheds from Australia, Ireland, New Zealand, the UK and Canada). The majority of studies were qualitative (12 papers), with 2 quantitative survey‐based studies and 2 mixed‐methods studies. Only two of the studies used validated health measures: the WHOQOL‐BREF scale [7] and the Beck Depression Inventory [5].

**Table 1:** A summary of papers included in the scoping review

Author and study	Country	Method of data collection	Sample size	Shed participants
Ayres, Patrick, and Capetola (2018). Health and environmental impacts of a regional Australian Men's Shed program. [1]	Australia	Qualitative: Semi‐structured individual and group interviews	1 shed (13 participants)	− Attendees of the shed and staff members − All male − Average age of 62 years
Ballinger et al. (2009). More than a place to do woodwork: a case study of a community‐based Men's Shed. [2]	Australia	Qualitative: In‐depth interviews	1 shed (8 participants)	− Attendees of the shed
Cordier and Wilson (2013). Community‐based Men's Sheds: promoting male health, well‐being and social inclusion in an international context. [3]	International	Quantitative	324 Australian and 59 international sheds representatives	− Representatives/ co‐ordinators of sheds − Gender unknown − Age range unknown
Crabtree et al. (2017). Men's sheds: the perceived health and well‐being benefits. [4]	UK	Qualitative: Semi‐structured interviews	2 sheds (8 participants)	− Attendees of the sheds − All male − All over 65 years
Culph, Wilson, Cordier, and Stancliffe (2015). Men's Sheds and the experience of depression in older Australian men. [5]	Australia	Qualitative: Semi‐structured interviews and observations	3 sheds (12 participants)	− Attendees of the sheds − All male − Average age of 67 years
Fildes et al. (2010). Shedding light on men: The building healthy men project. [6]	Australia	Qualitative: Participatory Action Research with semi‐structured interviews and journals	1 shed (15 participants)	− Attendees of the shed
Ford, Scholz, and Lu (2015). Social shedding: Identification and health of men's sheds users. [7]	Australia	Quantitative: Survey using WHOQOL‐BREF tool	332 responses from multiple Australian sheds (undisclosed)	− Attendees of the sheds − All male − Age range 25–86 years
Foster, Munoz, and Leslie (2018). The personal and community impact of a Scottish Men's Shed. [8]	UK	Mixed Methods: Survey and informal conversations	1 shed (31 participants)	− Attendees of the shed − All male − Average age of 69.7 years
Hansji, Wilson, and Cordier (2015). Men's Sheds: enabling environments for Australian men living with and without longterm disabilities. [9]	Australia	Qualitative: Semi‐structured interviews and observations	1 shed (12 participants)	− Attendees of the shed − All male − Age range 23‐85 years
Henwood et al. (2017). Men's health and communities of practice in Australia. [10]	Australia	Qualitative: Semi‐structured interviews and focus groups	5 sheds (61 participants)	− Shed leaders and co‐ordinators − All male − Age range unknown
Lefkowich and Richardson (2016). Men's health in alternative spaces: exploring men's sheds in Ireland. [11]	Ireland	Qualitative: Semi‐structured interviews, focus groups and observations	5 sheds (27 participants)	− Attendees of the shed − All male − Age range early 20s to mid‐70s
McGeechan et al. (2016). Exploring men's perceptions of a community‐based men's shed programme in England [12]	UK	Qualitative: Focus groups	19 sheds (32 participants)	− Attendees of the sheds − All male − Age range 18–69 years
Moylan et al. (2015). The Men's Shed: Providing biopsychosocial and spiritual support. [13]	Australia	Qualitative: Semi‐structured interviews and observations	1 shed (21 participants)	− Attendees of the shed − All male − Age range 18–91 years
Munoz, Farmer, Winterton, and Barraket (2015). The social enterprise as a space of well‐being: an exploratory case study. [14]	Australia	Qualitative: Walking interviews, observations and digital mapping	1 shed (24 participants)	− Attendees of the shed and staff members − All male − Age range unknown
Ormsby et al. (2010). Older men's participation in community‐based men's sheds programmes. [15]	Australia	Qualitative: Semi‐structured interviews	2 sheds (5 participants)	− Attendees of the sheds
Waling and Fildes (2017). ‘Don't fix what ain't broke’: evaluating the effectiveness of a Men's Shed in inner‐regional Australia. [16]	Australia	Mixed Methods: Survey and semi‐structured interviews	1 shed (22 participants)	− Attendees of the shed − All male − Age range 40–75 years

As demonstrated, most of the qualitative studies are characterised by small sample sizes, typically only collected in one shed. For example, Ormsby et al [15] completed semi‐structured interviews with only five respondents from two sheds. Studies from Lefkowich and Richardson [11] and McGeechan et al [12] collected data from participants across five sheds. Considering methodological approaches, however, McGeechan et al used only five focus groups, compared to Lefkowich and Richardson's more robust triangulation of data from interviews, focus groups and observations. Of the larger scale quantitative studies of Cordier and Wilson [3] and Ford et al [7], evidence is presented in both cases from over 300 Men's sheds; however, the studies have low‐response rates to surveys from each individual shed. The quantitative and qualitative mixed‐methods studies [8, 16] have triangulated data from small samples within only one shed. Further, only one longitudinal study was identified, that set out to measure changes in health and well‐being at three significant points over a two‐year period [6].

### 3.2 Demographic findings

Shed member samples from each study were typically described as retired or unemployed, with an age range of 18–91 years. Some of the sheds described the attendance or targeting of specific subgroups. This included those who were unemployed [3, 6, 10], had physical or learning disabilities [6, 9, 14], addiction issues [2, 10], previous criminal convictions [10, 12] and people with mental health issues [3, 5].

Of the 16 selected studies, 4 were specifically related to the health and well‐being of those who may already be classed as ‘at risk’ or already have existing health ailments [4, 5, 9, 15]. Two studies looked only at the health and well‐being of older participants over 65 years [4, 15]: one studied men with long‐term disabilities [9] and one study was directed at shed members with self‐reported or diagnosed depression [5].

Specific demographic information was gathered from participants in 14 of the 16 studies, including: age [1, 2, 5, 6, 7, 8, 9, 11, 12, 13, 15, 16], employment status [1, 2, 5, 6, 7, 8, 9, 11, 13, 14, 15], living conditions [2, 4, 7, 8, 15], educational attainment level [4, 7, 9] and marital status [4, 6]. Seven of the studies referenced information related to the geographical context of the study or levels of deprivation in the locality of the shed [3, 5, 6, 9, 10, 12, 14]. Out of the 16 studies, 4 described relationships between demographic variables (usually age) and health and well‐being outcomes [4, 5, 8, 15]. Men over the age of 60 years were more likely than younger men to report a sense of improved health and well‐being through increased physical capacity [4, 5] and a development of new companionship that had been lost through widowhood or loss of friends [15]. Similarly, factors correlated with older age and being retired, such as a loss of purpose, lack of opportunities to share knowledge and skills with others, were counteracted by shed activities [5, 15].

Evidence from the articles tentatively suggests that attending a shed may positively impact upon family life and relationships at home [6, 11, 13, 15, 16]. Nevertheless, very few studies collected household information of participants (living situation, marital status), and only one study [6] collected data from family/household members, which may have served to strengthen this line of enquiry. Two of the studies [3, 10] outlined demographic variables related to the context or location of the sheds (e.g. rurality, levels of deprivation). Cordier and Wilson [3] found that sheds in remote areas tended to be smaller in size with less shed members; however, remote sheds received the most visits from health workers delivering information and advice to shed members. Yet, this study did not comment on the potential links to improved health and well‐being as a result of such visits. The study also found a large proportion of sheds that responded to their survey were predominantly targeted towards vulnerable communities with high levels of unemployment and deprivation. Only one study directly discussed the health inequalities that shed members faced as a result of living in an area of deprivation stating that shed activities helped men to confront everyday issues related to unemployment, violence and substance use inherent to their community [10].

### 3.3 Development of logic model

**Figure 2:**
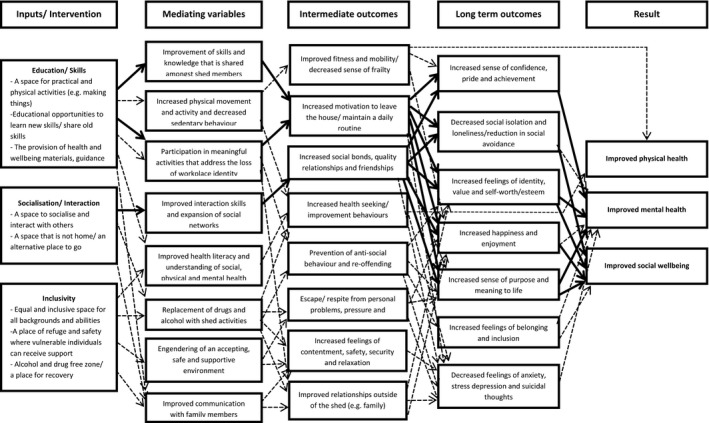
A logic model of the health and well‐being outcomes of Men's Shed Activities reported from 16 studies. Thicker lines = more than 10 papers reported this outcome; dotted lines = less than 10 papers reported this outcome

Figure 2 presents the logic model.

#### 3.3.1 Inputs/Intervention

The types of inputs described in each study were synthesised and three themes were identified: education and skills; socialisation and interaction; and inclusivity. The types of inputs and activities outlined in each of the shed studies identified in the review are shown in Table 2.

**Table 2:** Summary of the types of inputs described in each study

Education/ Skills	Socialisation/Interaction	Inclusivity
‘Work‐like’ spaces for woodwork and metalwork Opportunities to speak to healthcare professionals/learn about health Provision of health literature on physical and mental health	Space provided specifically for fostering socialisation with other shed members Space for events and social gatherings	Activities promoting an equal and inclusive space of acceptance Disability and mental health support contracts/arrangements Alcohol and drug free zones Provision of health and well‐being support A place of refuge and safety and recovery

All of the sheds included in the review followed a similar model for activities that can be described as *practical*, *educational* and *social*, where work‐like activities and socialisation co‐exist. Practical activities described included woodwork, metalwork, craft sessions, gardening, and maintenance and repair tasks. Although such activities provided a structure for ‘informal mentoring’ and the sharing of practical knowledge and experience [9:277], sheds also offered specific educational classes and training. The latter included visits from health professionals and access to health promotion materials such as health and safety training, manual handling courses or the promotion of weight management [11]. Sheds were found to provide assigned social areas where men could ‘simply meet and enjoy being in each other's company’, such as a seating area or kitchen [15:6]. The emphasis being the provision of an alternative space for men to congregate and relax, rather being ‘stuck at home’ [11:5].

#### 3.3.2 Mediating Variables

Mediating variables are outlined to give a clearer understanding of the relationship between the inputs of the sheds and the health and well‐being outcomes reported.

##### Education and skills

The most commonly reported mediating factor for the provision of education and skills was the opportunity for shed members to share and develop their practical skills, such as carpentry or metalwork [2, 8, 11, 14, 15, 16]. The latter helped to facilitate social interaction with others and the expansion of social networks through common interests. Moreover, skills and knowledge from previous employment could be continued in a meaningful and valued way. Less‐reported mediating variables were an increase in physical movement, such as walking or lifting caused by the involvement in practical activities [2, 4, 14]; and improved health literacy as a result of receiving education and advice on health and well‐being concerns from health professionals [3, 10]. Two papers [13, 15] suggested that the provision of practical activities and educational opportunities gave shed members more to talk about at home with their families.

##### Socialisation and interaction

The provision of space for socialisation and interaction predominantly provided the opportunity for shed members to improve their interaction skills and widen their social networks through meeting new people. In particular, Men's Sheds were identified as a socially acceptable place for men to meet other men while taking part in meaningful masculine activities [2, 4, 5, 9]. One study also reported that having the opportunity to socialise and interact with others had an effect on life at home, with storytelling between shed members being relayed to family members [15].

##### Inclusivity

Studies showed that sheds provided a supportive and inclusive environment for men of all backgrounds to attend, including those with physical or mental health issues [4, 5, 6, 11, 12, 13, 14, 15, 16]. It was found that this led to the mixing of shed members culturally and intergenerationally [2, 13, 14], and the integration of those with physical and learning disabilities [3, 9, 14] and mental health issues [2, 5, 10, 14]. Studies showed that as a result of having an inclusive and supportive environment, men were attending the shed rather than drinking alcohol or taking drugs outside of the shed [2, 10, 14].

#### 3.3.3 Intermediate and long‐term outcomes

Thematic analysis was used to refine the intermediate and long‐term health and well‐being outcomes that were identified in the literature into key themes (Thomas & Harden, 2008). This process involved sorting the inputs and health and well‐being outcomes into themes based on their meaning, for example, grouping of the terms with similar meanings like ‘social bonds’, ‘friendship’ and ‘meaningful relationships’. The most commonly mentioned themes were ranked, as presented in Table 3.

**Table 3:** The synthesis of intermediate and long‐term health and well‐being outcomes

Intermediate health and well‐being outcomes	Number of articles that reported this outcome
Increased social bonds, quality relationships and friendships	11
Increased motivation to leave the house/ maintain a daily routine	9
Increased feelings of contentment, safety, security and relaxation	5
Improved fitness and mobility/ decreased sense of frailty	4
Escape/ respite from personal problems, pressure and responsibility	4
Increased health seeking/ improvement behaviours	3
Prevention of antisocial behaviour and re‐offending	3
Improved relationships outside of the shed (e.g. family)	3

Long‐term health and well‐being outcomes	Number of articles that reported this outcome
Increased sense of purpose and meaning to life	11
Increased happiness and enjoyment	10
Increased sense of confidence, pride and achievement	9
Decreased social isolation and loneliness/reduction in social avoidance	9
Increased feelings of identity, value and self‐worth/esteem	9
Increased feelings of belonging and inclusion	4
Decreased feelings of anxiety, stress, depression and suicidal thoughts	3

Health and well‐being outcomes described in each study were organised into dimensions of physical health, mental health and social well‐being as based on the World Health Organization definition of health (WHO, 1948). Although such distinct components of health are not always mutually exclusive and typically overlap, these themes have been interpreted separately to display a clearer picture of the health and well‐being outcomes produced by sheds. In this review, both physical and mental health are related to the status of the individual, whereas social well‐being is being described in terms of ways of living together with others, and was in which an individual is included in groups, community or wider society (Keyes, 1998).

Studies that only inferred tentative causal links between shed activity and health and well‐being were included if such links had been shown in wider literature. For example, a study of a Men's Sheds implied that a decrease in alcohol use in shed members could potentially lead to a decrease in instances of violence, however there was no evidence to show this [10]. Therefore, this study was included as links between alcohol use and violence have already been widely substantiated in public health literature (Bellis & Hughes, 2011; Graham & Livingston, 2011).

##### Physical health

**Figure 3:**
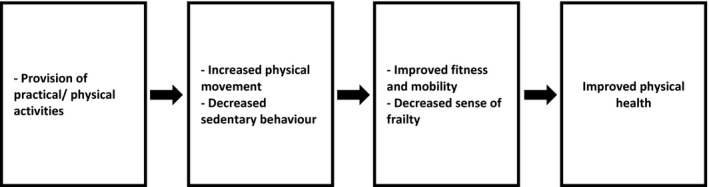
An observed health pathway leading to improved physical health

There were five studies where shed members stated any physiological health outcomes, and all of the data was self‐reported [1, 4, 10, 13, 14]. The outcomes described frequently related to the improved physical health through increased in physical activity and decreased sedentary behaviour (shown in Figure 3). For instance, reported studies referred to increased movement and levels of fitness through leaving the house and taking part in manual labour‐like activities. Crabtree et al [4] found that shed members were walking and standing more while at sheds, which had led to increased mobility and decreased feelings of frailty; this was not mentioned in any other studies.

A small number of studies reported that shed members decreased their alcohol and drug consumption as a result of being in a sober shed environment [10, 14]; the long‐term physical (or mental) health outcomes of this abstinence were not studied. With sheds described as an inclusive space to manage addiction to substances [2], this particular area of preventative health is still relatively unexplored. Cordier and Wilson [3] found that due to health professionals visiting sheds, there was an increase in health literacy among shed participants; nevertheless, it was unclear whether increased health‐related knowledge led to improvements in physical health.

##### Mental health

**Figure 4:**
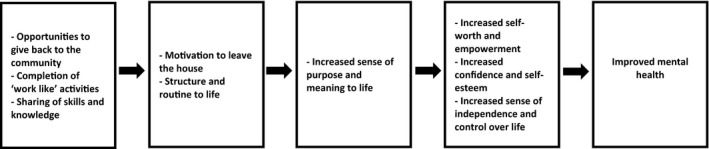
An observed health pathway leading to improved mental health

The most commonly reported mental health outcome, mentioned in all 16 of the studies, was an increased sense of purpose and meaning to individual participants lives. This outcome was mainly a result of having a reason to leave the house and the addition of structure and routine to their lives, which gave them a meaningful place in society. A potential pathway to improved metal health is presented in in Figure 4.

An increased sense of purpose was commonly reported by those who were unemployed, retired or ex‐prisoners, and those who had lost their ‘work identity’ [11:4]. Activities that contributed to a sense of purpose included an opportunity to help others and ‘give back’ to the community [14, 15], participate in masculine ‘work‐like’ activities that mimicked employment [2, 4, 14], and being able to utilise and share skills and knowledge with others [11]. Having a sense of purpose and meaning was found to lead to increased feelings of self‐worth and empowerment [14], increased sense of masculine identity [2, 4, 5, 9], confidence and self‐esteem [8, 9] and a sense of independence and control in life [13].

Decreased depression and instances of suicidal thoughts was mentioned as an outcome of shed activities in 10 of the 16 studies, however, only by a small number of people within each sample [2, 4, 5, 8, 9, 10, 11, 14, 15, 16]. This was related to having a safe place for mental recovery from trauma or addiction [10, 11, 14], keeping active and engaged in an activity and getting out of the house [2, 15], having an increased sense of self‐efficacy, hardiness and resilience [4, 5], being able to share problems and experiences with others, and gain social support [9, 10].

Further outcomes that may lead to improved mental health were an increased sense of pride, achievement and satisfaction from producing things of value, such as woodwork [4, 6, 15], and an increased confidence through the development of skills and educational opportunities [8, 10]. Again, these areas were not investigated in detail.

##### Social well‐being

**Figure 5:**
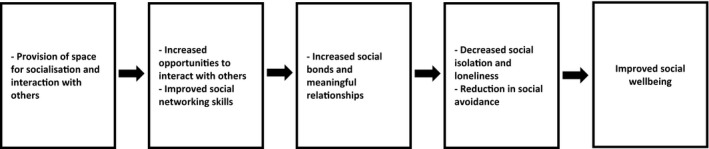
An observed health pathway leading to improved social well‐being

A common social well‐being outcome described in 14 of the studies was an increase in social networks and interactions as a result of attending a shed and taking part in activities [not mentioned in papers 3 and 7]. A greater sense of belonging from increased social networks was reported in seven of the studies [1, 2, 8, 9, 10, 11, 14]. Most notably, the term ‘social inclusion’ was described in relation to the integration of participants with disabilities, addictions and criminal records, who may previously have felt marginalised from society [8, 10, 11]. Social support and solidarity from other shed members, the engendering of a safe and accepting environment, and the ability to share experiences of vulnerability were reported to increase the feeling of being included in a community [12, 14, 16]. In five of the studies, increased social networks and social inclusion was reported as leading to a decreased sense of social isolation, as shown in Figure 5 [4, 8, 11, 12, 13].

A small proportion of studies described indirect social well‐being outcomes related to the effect that participation in sheds might have on their lives outside of the shed. Only one of the studies [6] conducted interviews with family members, therefore, the findings were predominantly anecdotal. Shed members reported sharing stories of their day at the shed with family members, which provided an interesting discussion point [13, 15]. Furthermore, participation in sheds allowed men to contribute to the household in the form of *‘goods, skills and social interaction’* [13:226], leading to improved happiness and sense of purpose at home, particularly among retired men [6]. Waling and Fildes [16] found that as a result of attending a shed, men found an outlet for their stress and anger, leading to a decrease in arguments and aggression at home.

## 4 DISCUSSION

This paper developed a plausible causal theory and a logic model showing how Men's Sheds may influence the health and well‐being of their participants. The study has shown that although sheds have been in existence since the 1990s; there is a lack of reliable and systematic evidence of the health and well‐being outcomes of shed activities.

We observe that very few of the studies included in our review collected demographic information on the shed participants further than age and gender. Associative relationships between factors such as employment status or marital status and health and well‐being outcomes are under‐explored. Furthermore, few studies have investigated the health and well‐being outcomes reported within differing groups that are using sheds, such as those with disabilities or addictions. The links between shed activity and the indirect or secondary effects on wider populations, such as family members, has been inferred in one study (Fildes, Cass, Wallner, & Owen, 2010); again, this area of shed member's lives has had little consideration. There has also been a lack of attention of the contextual variables of sheds, such as aspects of rurality or levels of deprivation, and the subsequent links to existing health inequalities within communities. These contextual factors are important to locate each shed in a specific place and space, and to allow for the consideration of variable differences in individuals and communities. Although largely qualitative or based on small samples, a body of evidence describes the plausible mental and social well‐being impacts of shed activities; yet, less is known about the impacts on physical health.

Evidence that exists on the links between shed activities and health and well‐being relies on self‐reported data from small sample sizes, with a deficit of quantitative or mixed method studies that use validated health outcome measures. Furthermore, only one study captured health and well‐being changes over a significant period to allow for comparisons (Fildes et al., 2010). Subsequently, existing knowledge is limited in its generalisability. The latter has an impact on the presented theory and a logic model, which captures currently limited and scarce findings. The logic model remains limited by the nature of the data available on this specific topic, and the inclusion criteria adopted that excluded further evidence from non‐peer‐reviewed documents. Furthermore, the quality of the included papers was not assessed, which may have provided a standard of evidence for meaningful and robust results. To test and further develop the proposed theory and the logic model, and evidence impact of sheds on their participants, we call for more research and suggest broad future research directions.

In order to address research gaps, there is a requirement for larger scale quantitative and mixed‐method studies for a more robust picture of Men's Sheds as a potential health intervention. Future studies would benefit from a longitudinal approach to measure changes in shed user's health and well‐being over a longer time period. Furthermore, the use of validated health and well‐being measures are needed for the validity and replicability of the findings, and for future evaluations to compare Men's Sheds with other interventions for improving health and well‐being.

The diversity of sheds and their participants has yet to be explored in relation to place and context (e.g. cultural and geographical background), as well as among different subgroups of users with long‐term health ailments and disabilities. Although some studies touched upon cultural factors relating to gender, further attention is required to the broader social context of male culture and masculine identity and how this may shape the way that Men's Shed activities impact on individuals’ health and well‐being. With studies predominantly reporting mental health and social well‐being outcomes, evidence about physical health outcomes is needed that moves beyond self‐reported data. In particular, this study suggests that research would usefully focus on health benefits related to decreased sedentary behaviour, and a decrease in alcohol and drug use as a result of visiting a shed. Future studies should also include a comparator group to validate generated findings.

## 5 CONCLUSION

The review has identified the literature that exists on Men's Sheds and health and well‐being at this point in time, where sheds are still a developing concept. What has been produced represents a logic model with a clear visual indication of the possible directions of causality between shed activities and health and well‐being. This allows for the potential formation of hypotheses on the ability of sheds to generate health in a way that is gender specific, and that caters for their specific needs and sensitivities.

## CONFLICT OF INTEREST

No conflicts of interest have been declared.

## ENDNOTE

1 For more detailed studies on masculinity and male identity, see Connell (2005) and Remy (1990).
